
JOURNAL INFORMATION
==============================
NLM Title Abbreviation: Medizinrecht
Journal ID: Medizinrecht
NLM Journal ID: 8309354

Medizinrecht

ISSN: 0723-8886
EISSN: 1433-8629

ARTICLE INFORMATION
==============================
PMCID: PMC9432799
PMID: 36065299
DOI: 10.1007/s00350-022-6292-9
Article ID: 6292
Article version: 1
Subjects: Aufsätze

Wissensgenerierung zur Pandemievorsorge und -steuerung durch (digitale) Public Health Surveillance

Kuhlmann Simone
grid.9026.d 0000 0001 2287 2617 Zentrum für Recht in der digitalen Transformation, Rechtswissenschaftliche Fakultät, Universität Hamburg, Von-Melle-Park 5, 20148 Hamburg, Deutschland
Electronic publication date: 2022 Aug 31
Print publication date: 2022
Volume: 40
Issue: 9
First page: 730
Last page: 736
Copyright: © Der/die Autor(en) 2022
License: Open Access Dieser Artikel wird unter der Creative Commons Namensnennung 4.0 International Lizenz veröffentlicht, welche die Nutzung, Vervielfältigung, Bearbeitung, Verbreitung und Wiedergabe in jeglichem Medium und Format erlaubt, sofern Sie den/die ursprünglichen Autor(en) und die Quelle ordnungsgemäß nennen, einen Link zur Creative Commons Lizenz beifügen und angeben, ob Änderungen vorgenommen wurden.
Die in diesem Artikel enthaltenen Bilder und sonstiges Drittmaterial unterliegen ebenfalls der genannten Creative Commons Lizenz, sofern sich aus der Abbildungslegende nichts anderes ergibt. Sofern das betreffende Material nicht unter der genannten Creative Commons Lizenz steht und die betreffende Handlung nicht nach gesetzlichen Vorschriften erlaubt ist, ist für die oben aufgeführten Weiterverwendungen des Materials die Einwilligung des jeweiligen Rechteinhabers einzuholen.
Weitere Details zur Lizenz entnehmen Sie bitte der Lizenzinformation auf http://creativecommons.org/licenses/by/4.0/deed.de.
License URL: https://creativecommons.org/licenses/by/4.0/

issue-copyright-statement: © Springer-Verlag GmbH Deutschland, ein Teil von Springer Nature 2022

==============================

**Simone Kuhlmann**

Die COVID-19-Pandemie hat offenbart, welche zentrale Rolle die Kenntnis über die gegenwärtige Gesundheitslage zur Handlungssteuerung und Vorsorge spielt und wie wichtig dafür ein kontinuierliches datenbasiertes Abbild der Versorgungssituation ist. Einschneidende Public-Health-Maßnahmen von Kontaktbeschränkungen, über Schließung von Schulen und Einrichtungen bis hin zu Ausgangs- und Reisebeschränkungen wurden und werden auf Basis und in Abhängigkeit von Modellierungen und Simulationen auf Basis von Hospitalisierungsraten, Fallzahlen je 100.000 Einwohner oder der Belegung von Intensivbetten getroffen – obwohl vor allem in Deutschland häufig valide Daten fehlen[1]. Zwar billigt das BVerfG dem deutschen Gesetzgeber auch bei bestehender Unsicherheit, was die tatsächlichen Verhältnisse anbelangt, die Möglichkeit zum Handeln und Ergreifen selbst einschneidender Maßnahmen zu – vor allem wenn es um den Schutz gewichtiger Verfassungsgüter geht[2]. Dieser Spielraum reicht aber stets nur soweit, wie es dem Gesetzgeber nicht möglich ist sich ein hinreichend sicheres Urteil zu bilden und für einen Erkenntnisfortschritt zu sorgen[3]. Besteht also die Möglichkeit fundierte Erkenntnisse zu erlangen, so lassen sich Maßnahmen nicht mehr allein auf reine Prognosen und Annahmen stützen. Abgesehen davon, dass es dem Gesetzgeber zur Legitimation seiner Maßnahmen ohnehin stets darauf ankommen sollte, Entscheidungen auf eine solide und valide Datenbasis zu stellen, sind Einschränkung individueller Freiheitsrechte doch nur solange gerechtfertigt, wie sie angesichts der tatsächlichen Lage und ihrer Wirkung überhaupt (noch) zweckdienlich sind.

### I. Puplic Health Surveillance als Basis kontinuierlicher Wissensgenerierung

Wesentlicher Baustein zur Gewinnung einer kontinuierlichen und validen Datenbasis über die Gesundheit der Bevölkerung – auch jenseits akuter Lagen – ist die sog. Public Health Surveillance (PHS). Sie beschreibt die kontinuierliche, systematische Erhebung, Analyse, Interpretation und Berichterstattung von gesundheitsbezogenen Daten für die Planung, Implementation und Evaluation von Maßnahmen im Bereich Public Health[4]. Ihr Ziel ist dabei nicht allein die frühzeitige Erkennung potentieller, akuter Gesundheitsgefahren (wie Krankheitsausbrüche, Epidemien, Bioterrorismus etc.) und deren Überwachung, um zeitnahe Reaktionen des Gesundheitswesen zum Schutz der Bevölkerung zu ermöglichen (sog. Infektionsepidemiologische Surveillance). Auch das Monitoring und die Aufklärung

1) Vgl. dazu 5. Stellungnahme des ExpertInnenrates der BReg. zu COVID-19 v. 31. 1. 2022; insb. zur fehlenden Datenlage während der Omicron-Welle 2021/2022 vgl. Interview *G. Antes* v. 6. 1. 2022 unter https://www.t-online.de/nachrichten/deutschland/id_91431034/corona-datenlage-karl-lauterbach-koennte-voellig-danebenliegen-.html (zuletzt abgerufen am 3. 8. 2022).
2) BVerfG, Beschl. v. 19. 11. 2021 – 1 BvR 781/21 –, Rdnr. 185.
3) Vgl. BVerfG, Beschl. v. 19. 11. 2021 – 1 BvR 781/21 –, Rdnrn. 185, 190; BVerfGE 50, 290, 332 f.; BVerfGE 88, 87, 97.
4) World Health Organization (2022), surveillance in emergencies, https://www.who.int/emergencies/surveillance (zuletzt abgerufen am 3. 8. 2022); *Reitzle et al.*, Bundesgesundheitsbl. 2020 63:1099; *Ziese/Hamouda*, Gesundheitswesen 2017:79, 932, 933.

der Epidemiologie von Gesundheitsproblemen, wie etwa Krebs, Diabetes oder der psychischen Gesundheit, zur gezielten (auch präventiven) Bekämpfung von Leiden oder die Erbringung von Nachweisen über die Wirksamkeit von Interventionen sind in den letzten Jahren zu wesentlichen Bestandteilen der Public Health Surveillance geworden[5], sollen jedoch für diesen Beitrag außer Betracht bleiben.

Vor allem was die für die Pandemievorsorge wichtige infektionsepidemiologische Überwachung betrifft, erfolgte die PHS bis zur Corona-Krise – vor allem hierzulande – in aller Regel noch herkömmlich, über Meldeketten, basierend auf regional manuell gesammelten medizinisch verifizierten Daten (Labor- und Falldaten etc.), ohne automatisierte, unmittelbar zentralisierte Sammlung – mit all den sich dabei offenbarenden Defiziten der Unvollständigkeit und zeitlichen Verzögerung. Für viele Fragestellungen der öffentlichen Gesundheit, wenn es bspw. um die langfristige Gestaltung der Versorgung geht, mag dies akzeptabel sein, in zeitkritischen Situationen wie denen einer Pandemie bedarf es hingegen einer kontinuierlich aktuellen und möglichst breiten datenbasierten Wissengrundlage, um eine evidenzbasierte und zielgerichtete Handlungssteuerung der Verantwortlichen zu ermöglichen.

Geliefert werden kann diese Datengrundlage in Zeiten, in denen immer mehr elektronische Versorgungsdaten verfügbar sind, unter Zuhilfenahme digitalen Surveillance-Tools, die die Daten im Idealfall automatisch und nahezu in Echtzeit an den unterschiedlichen Stellen und Akteuren des Gesundheitssystems sammeln und mittels statistischer Verfahren Künstlicher Intelligenz auf Muster, Zusammenhänge etc. analysieren[6]. Dabei lassen sich nicht nur wie bisher primär zu Surveillance-Zwecken erhobene Daten, wie Fallzahlen meldepflichtiger Krankheiten, Zahl der Krankenhauseinweisungen oder jüngst auch Abwasserdaten[7], nutzbar machen. Auch sog. Sekundärdaten, die mit einer anderen Zielsetzung erhoben werden und vielfach in großen Mengen kontinuierlich – z. T. sogar frei verfügbar – vorliegen, wie bspw. Social Media- und Mobilfunkdaten oder Daten aus den Gesundheits- und Sozialversicherungssystemen (Kranken-, Renten-, Unfallversicherung), weisen ein nicht zu verachtendes Potential für die PHS auf[8]. So kann die ergänzende Analyse solcher Sekundärdaten nicht nur die Möglichkeit erhöhen, potentielle Krankheitsausbrüche und sonstige Gesundheitsgefahren frühzeitiger – jedenfalls vor ihrer Manifestation im Gesundheitssystems – zu erkennen[9]. Sie können auch etwaige Unvollständigkeit und Verzerrung durch Einbeziehung von Personengruppen, die in den herkömmlichen Gesundheitsdaten nicht abgebildet werden, bspw. weil sie einen schlechteren Zugang zur Gesundheitsversorgung haben, verringern.

Vor diesem Hintergrund untersucht der Beitrag die rechtliche und administrative Ausgestaltung von Surveillance-Systemen auf nationaler und europäischer Ebene unter besonderer Berücksichtigung bereits implementierter digitaler Komponenten und Wissensinfrastrukturen. Im Fokus stehen dabei insb. der deutsche und europäische Rechtsrahmen zur Bekämpfung übertragbarer Krankheiten, einschließlich dem Vorschlag der EU-Kommission zur Gesundheitsunion, der das bisherige europäische Surveillance-Instrumentarium grundlegend modernisieren soll.

### II. Surveillance-Systeme in Deutschland – rechtliche und administrative Ausgestaltung der infektionsepidemiologischen Überwachung

In Deutschland basiert die infektionsepidemiologische Überwachung im Wesentlichen auf zwei Säulen: Dem Meldewesen gemäß §§ 6 ff. IfSG sowie der syndromischen Surveillance (§ 13 Abs. 4 S. 2 Nr. 2 IfSG), welche durch die molekulare und virologische Surveillance (§ 13 Abs. 3 IfSG) ergänzt wird. Während ersteres meldepflichtige (Verdachts-) Fälle von Infektionskrankheiten und Krankheitserregern sowie deren Häufigkeit anhand klarer Falldefinitionen detailliert erfasst, ermittelt die syndromische Surveillance Krankheitsfälle anhand charakteristischer klinischer Krankheitszeichen, d. h. also entlang des Auftretens von Symptomen, die relativ spezifisch auf bestimmte Infektionskrankheiten hinweisen[10]. Gegenüber dem Meldewesen bietet das den Vorteil, dass auch das Auftreten und die Ausbreitung von bislang unbekannten Krankheiten und Krankheitserregern erkannt werden kann. So konnte bereits zu Beginn der COVID-19-Pandemie der Anstieg von Pneumonie beobachtet werden, noch bevor schwere Covid-19-Fälle offiziell in Deutschland in größerem Maße registriert wurden. Beide Surveillance-Systeme haben jedoch den Nachteil, dass sie nur auf Informationen von im Gesundheitssystem vorstelligen Personen zurückgreifen können und damit häufig nur die Spitze des Eisbergs abbilden. Entsprechend sieht das IfSG als weitere Formen der epidemiologischen Überwachung einerseits die Durchführung sog. Sentinel-Erhebungen (§ 13 Abs. 2 IfSG) vor, die die Häufigkeit der Verbreitung einer bestimmten Infektionskrankheit in der Gesamtbevölkerung stichprobenartig anhand einer ausgewählten Bevölkerungsgruppe bestimmen[11], andererseits lässt es weitere Instrumente, wie etwa Kohortenstudien, auf Basis der allg. Befugnisnorm § 13 Abs. 1 IfSG zu. Bei beiden Formen der Datenerhebung basieren hierbei – anders als im Rahmen des Meldewesens, wo eine gesetzliche Pflicht zur Meldung besteht – auf einer rein aktiven und freiwilligen Mitarbeit der beteiligten Akteure (Arztpraxen, Kliniken, Laboratorien, Bildungseinrichtungen, teilnehmende Personen), die die Daten erheben bzw. die Datenerhebung zulassen und an den verantwortlichen Träger weitergeben.

In der Praxis erfolgt die Surveillance jenseits des gesetzlichen Meldewesens anhand einer Vielzahl unterschiedlicher, von verschiedenen Akteuren des Gesundheitswesens und der Wissenschaft in der Regel in Zusammenarbeit mit dem Robert-Koch-Institut (RKI) getragener kaum zu überblickender Einzelprojekte. Exemplarisch sei hier etwa nur auf das Onlineportal *GrippeWeb*[12] zur Erfassung akuter Atemwegserkrankungen in der Bevölkerung durch die Arbeitsgemeinschaft Influenza (AGI)[13], das syndromische Krankenhaus-Surveillance-System *ICOSARI*[14], das das RKI in Kooperation mit der HELIOS Kliniken GmbH zur Surveillance schwerer akuter respiratorischer Erkrankungen (ARE) betreibt, oder auf den jüngst entwickelten Krankenhaus-Surveillance-Monitor *SUMO*[15], der allg. Routinedaten aus Notaufnahmen erfasst und ungewöhnliche Verän-

5) *Ziese/Hamouda*, Gesundheitswesen 2017:79, 932, 933.
6) Vgl. hierzu nur *Abad et al.*, Digital Public Health Surveillance: a systematic scoping review, npj Digital Medicine (2021) 4:41; *Bansal et al.*, Big Data for Infectious Disease Surveillance and Modeling, JID 2016:214.
7) https://www.zeit.de/news/2022-02/09/projekte-zur-coronavirus-ueberwachung-im-abwasser-starten (zuletzt abgerufen am 3.8.2022).
8) *Ziese/Hamouda*, Gesundheitswesen 2017:79, 932, 934.
9) Bspw. erkannte die von der Harvard Medical School entwickelte Surveillance Plattform *Health Map* im Jahr 2014 den bis heute größten Ebola Ausbruch in Westafrika durch automatisierte Analyse von lokalen Nachrichtenberichten und Berichten in sozialen Medien neun Tage bevor die WHO den Ausbruch bestätigte, vgl. dazu *Roberts*, EJRR 2019, 722, 732 m. w. N.
10) BT-Dr. 19/23944, S. 27.
11) Vgl. hierzu BT-Dr. 14/2530, S. 67 f.; *Putzer*, in: *Eckart/Winkelmüller*, Infektionsschutzrecht, 2020, § 13, Rdnr. 6.
12) https://grippeweb.rki.de/ (zuletzt abgerufen am 3.8.2022).
13) Hierbei handelt es sich um eine Gemeinschaft der Sentinel-Praxen und des RKI.
14) Vgl. *Buda et al.*, BMC Public Health 2017 17:612; *Goerlitz et al.*, Bundesgesundheitsbl. 2021, 399.
15) Vgl. hierzu *Grabenhenrich et al.*, Bundesgesundheitsbl. 2021, 416.

derungen automatisch detektiert, verwiesen[16]. Das RKI als für die Krankheitsbekämpfung und -prävention zuständiges „infektionsepidemiologisches Zentrum“, fungiert dabei als übergreifende Koordinierungs- und Informationsstelle[17]. Seine vorrangige Aufgabe in diesem Kontext ist es, die epidemiologische Surveillance durch Falldefinitionen und Datenaustauschformate zu systematisieren und strukturieren, die durch sie gewonnenen Daten zu analysieren, epidemiologisch zu bewerten sowie anschließend über die gewonnenen Erkenntnisse zu informieren.

Die verschiedenen existierenden Surveillance-Systeme arbeiten dabei inzwischen vielfach unter Zuhilfenahme digitaler z. T. automatisierter Tools und Verfahren, die – auch aufgrund der jeweils unterschiedlich beteiligten Akteure – in der Regel eigens für den Zweck entwickelt worden und bislang in sich geschlossene Systeme mit jeweils eigener Betriebskultur sind. So nutzt etwa die AGI zur syndromischen Überwachung von ARE neben *GrippeWeb* das elektronische Erfassungssystem SEED$^{ARE}$, das in beteiligten primärversorgenden Arztpraxen anhand der ICD-10-Codes (International Statistical Classification of Diseases and related Health Problems, 10th revision) automatisiert die im Arztinformationssystem ASI hinterlegten Falldaten[18] anonymisiert erfasst[19]. Das 2015 gestartete und ebenfalls für die ARE-Surveillance eingesetzte und auf Basis von ICD-10-Codes arbeitende Krankenhaus-Surveillance-System *ICOSARI* überträgt die anonymisierten Falldaten hingegen wöchentlich über einen eigenen Webserver des Rechenzentrums der HELIOS Kliniken an das RKI[20]. Beide Systeme laufen also, obwohl sie denselben Zweck verfolgen und anhand derselben Marker (ICD-10-Codes) arbeiten, parallel mit der Folge, dass die Daten nicht automatisch zusammengeführt werden, sondern in der Zusammenschau manuell ausgewertet werden müssen. Letzteres geschieht zwar durch die AGI bzw. das RKI, welches die aus den verschiedenen Surveillance-Systemen gewonnenen Erkenntnisse in Wochenberichten veröffentlicht[21]. Ein großes, umfängliches, verschiedene Versorgungsebenen abbildendes Dataset, welches auch die weitere Nutzung zu anderen Zwecken, etwa zur Forschung, erlauben würde, liegt jedoch nicht vor.

Zurückführen lässt sich dieser Umstand insb. auch auf die Tatsache, dass es – abgesehen vom Meldesystem gemäß §§ 6 ff. IfSG – in Deutschland weitgehend an gesetzlichen Maßgaben und Regelungen fehlt, was die Einrichtung und den Betrieb von Surveillance-Systemen betrifft[22]. Zwar hat der Gesetzgeber mit der Novellierung des § 13 IfSG im Jahr 2017 erstmals eine gesetzliche Grundlage für weitere – z. T. bereits in der Praxis etablierte – Formen der epidemiologischen Surveillance neben den Sentinels geschaffen[23]. Die Entscheidung ihrer Durchführung obliegt aber ebenso wie ihre konkrete Ausgestaltung und Operationalisierung im Einzelnen nach wie vor den Gesundheitsbehörden bzw. dem RKI, vgl. § 13 Abs. 1 IfSG. So kann insb. das Bundesgesundheitsministerium festlegen, welche Krankheiten und Krankheitserreger überwacht werden und durch Rechtsverordnung die Durchführung bestimmter Surveillance-Maßnahmen, wie etwa die Ablieferung von Untersuchungsmaterial und Isolaten von Krankheitserregern anordnen (§ 13 Abs. 3 S. 8, Abs. 4 S. 2 IfSG)[24]. Diese Offenheit hat insb. mit Blick auf die Ungewissheit hinsichtlich des Auftretens und Entstehens neuer Krankheitserreger den Vorteil, dass situativ unkompliziert bedarfsspezifische Surveillance-Systeme eingerichtet werden können, ohne das es eines (zeitintensiven) Tätigwerdens des Gesetzgebers bedarf. Zugleich wird durch das Fehlen detaillierter rechtlicher Rahmenbedingungen etwa was die Festlegung von Standards für die Datenerfassung und deren Aufbereitung oder auch das Bereithalten von Schnittstellen betrifft, jedoch die Chance vertan, langfristig eine Zusammenführung der verschiedenen Surveillance-Daten zu sog. Datenpools zu vereinfachen und zugleich Aspekte des Datenschutzes, der Betriebsverantwortung, Anforderungen an das System (z. B. IT-Sicherheit, Leistungsfähigkeit), und der Beteiligung zu regeln oder auch den Zugang zu den dort gesammelten Daten durch Zugangsrechte zu steuern[25]. Besonders letzteres erscheint mit Blick auf die Ermöglichung weiterer Forschung auch durch Dritte sinnvoll.

Rechtliche Maßgaben für den Betrieb eines digitalen Surveillance-Systems in Deutschland finden sich bislang ausschließlich im § 14 IfSG, der die Einrichtung eines elektronischen Melde- und Informationssystems (DEMIS) unter Verantwortung des RKI vorsieht. Das System soll seinem Zweck nach vor allem der schnelleren und effektiveren Übermittlung der durch die meldepflichtigen Stellen erhobenen Daten an die zuständigen Behörden und deren automatischen Abgleich dienen (§ 14 Abs. 3 IfSG); darüber hinaus aber auch die automatisierte Analyse der verarbeiteten Daten auf ein gehäuftes Auftreten einer Infektionskrankheit, bei denen ein epidemischer Zusammenhang wahrscheinlich ist, ermöglichen (§ 14 Abs. 5 IfSG). Auch wenn dadurch auf den ersten Blick gesetzlich ein digitales Betriebssystem zur Surveillance etabliert wird, welches langfristig breit einschließlich für die Übermittlung und Analyse der mit Hilfe anderer Formen epidemiologischer Surveillance (§ 13 IfSG) gewonnener Daten (§ 14 Abs. 2 Nr. 3 IfSG) eingesetzt werden kann, scheitert dessen Nutzbarmachung indes bislang an der fehlenden technischen Umsetzung. Zwar ist im Zuge der COVID-19-Pandemie der Ausbau von DEMIS erheblich vorangetrieben worden mit dem Ergebnis, dass inzwischen alle Gesundheitsämter und ein Großteil der Labore angeschlossen sind und Laborergebnisse elektronisch an die zuständigen Gesundheitsämter übermittelt werden[26]. Nichtsdestotrotz steckt das seit 2016 in der Entwicklung befindliche System noch weitgehend in seinen Kinderschuhen. So konnten bislang (Stand 21. 12. 2021) ausschließlich SARS-CoV-2 Erregernachweismeldungen elektronisch über das System übermittelt werden[27]. Andere Meldepflichtige, wie bspw. Arztpraxen oder sonstige in die Meldekette eingebundene Einrichtungen wie das RKI sind noch gar nicht angebunden, sondern sollen erst im Rahmen weiterer Ausbaustufen in das System integriert werden, so dass deren Meldungen bis auf weiteres herkömmlich über die etablierte traditionelle Infrastruktur per Fax oder Telefon erfolgen müssen. Das gilt insb. auch für die Übermittlung der aggregierten Falldaten an das RKI gemäß §§ 10 ff. IfSG, anhand derer das Institut

16) Für weitere Bsp. s. *Goerlitz et al.*, Bundesgesundheitsbl. 2021, 395 ff.
17) *Schmidt am Busch*, Die Gesundheitssicherung im Mehrebenensystem, 2007, S. 88 ff.; *Klafki*, Risiko und Recht, 2017, S. 199 f.; *Trute*, GSZ 2018, 125, 130.
18) Hierzu zählen Angaben zum Alter, Geschlecht, Influenzaimpfung in der Praxis, Arbeitsunfähigkeit, Hospitalisierung, Anzahl der Konsultationen in der Praxis.
19) *Goerlitz et al.*, Bundesgesundheitsbl. 2021, 398; *RKI*, Epidemiologisches Bulletin Nr. 30 v. 29. 7. 2021, Syndromische Surveillance von akuten Atemwegserkrankungen mit COVID-19 im ambulanten Bereich, S. 3.
20) *Buda et al.* Establishing an ICD-10 code based SARI-Surveillance in Germany – description of the system and first results from five recent influenza, BMC Public Health 2017 17:612), S. 2.
21) https://influenza.rki.de/ (zuletzt abgerufen am 3. 8. 2022).
22) *Grabenhenrich et al.*, Bundesgesundheitsbl. 2021, 416 f.
23) BT-Dr. 18/10938, S. 59.
24) Beispielhaft sei hier die Coronavirus-Surveillanceverordnung (CorSurV) genannt, die u. a. Laboratorien verpflichtet Daten zur vorgenommene Genomsequenzierung des Coronavirus an das RKI zu übermitteln.
25) Vgl. dazu jüngst auch das Positionspapier des Wissenschaftsrates, Digitalisierung und Datennutzung für Gesundheitsforschung und Versorgung, Juli 2022, S. 42 ff.
26) Vgl. zum Ausbau des Meldesystems detailliert *Diercke et al.*, Bundesgesundheitsbl. 2021, 388 ff.
27) *Diercke et al.*, Bundesgesundheitsbl. 2021, 390.

fortlaufend eine Bewertung der epidemiologischen Lage vornimmt und seinen völker- und unionsrechtlichen Meldepflichten nachkommt[28].

Begrüßenswert ist daher, dass mit dem § 14 Abs. 8 IfSG nunmehr bundesgesetzlich eine stufenweise in Kraft tretende Nutzungspflicht sowohl für Gesundheitsämter als auch für Meldepflichtige nach § 8 IfSG eingeführt worden ist, um langfristig eine einheitliche und zeitnah verfügbare elektronische Datenbasis zu etablieren[29]. Denn anders wäre wohl die auch aufgrund der föderalen Strukturen gewachsene Heterogenität der unterschiedlich eingesetzten Softwareprodukte in den Gesundheitsämtern kaum abzubauen.

### III. Public Health Surveillance auf EU-Ebene

Ebenso wie der deutsche Rechtsrahmen auf nationaler Ebene sieht auch das europäische Recht zur Überwachung übertragbarer Krankheiten ein Surveillance-System zur Krisenprävention auf europäischer Ebene vor, welches im Vergleich jedoch bereits über ein digitales Instrumentarium verfügt und nunmehr durch den Vorschlag der EU-Kommission zur Europäischen Gesundheitsunion[30] nochmals gestärkt und weitergehend digitalisiert werden soll. Grundlage dafür bildet der auf Basis von Art. 168 Abs. 5 AEUV ergangene Beschluss des Europäischen Parlaments und des Rates Nr. 1082/2013/EU[31] zu schwerwiegenden grenzüberschreitenden Gesundheitsgefahren (EU Decision on Serious Cross-border Threats to Health), welcher verbindliche Maßnahmen[32] zur Beobachtung, frühzeitigen Meldung und Bekämpfung derartiger Gefahren festlegt. Aufgrund der lediglich komplementären Zuständigkeit der Union im Bereich des Gesundheitsschutzes können diese Maßnahmen entsprechend Art. 2 Abs. 5 i. V. mit Art. 6 S. 2 lit. a) AEUV jedoch nur ergänzender, unterstützender und koordinierender Natur bleiben[33]. Eine Angleichung der national gesetzlich normierten Kriseninterventionsstrukturen durch europäischen Vorgaben ist ausgeschlossen; vielmehr bleiben die Mitgliedstaaten trotz der erkennbar transnationalen Problematik der Pandemiebekämpfung aufgrund der Komplementärzuständigkeit der EU „Herren der Gesundheitspolitik“[34], was während der COVID-19-Pandemie vor allem bei operativen Maßnahmen deutlich zutage getreten ist.

#### *1. Instrumente der epidemiologischen Überwachung*

Kernelement der unionalen Krisenpräventionsstrukturen bildet gemäß Art. 6 des Beschlusses Nr. 1082/2013/EU das Netz zur epidemiologischen Überwachung übertragbarer Krankheiten und damit zusammenhängender besonderer Gesundheitsrisiken biologischen Ursprungs (mit Ausnahme von Biotoxinen), welches den permanenten Austausch zwischen der EU-Kommission, der europäischen Agentur ECDC sowie den in den Mitgliedstaaten zuständigen Behörden sicherstellen soll[35]. Zuständig und verantwortlich für das Netz ist das European Centre for Disease Prevention and Control (ECDC), welches 2005 nach dem weltweiten SARS-CoV-Ausbruch im Jahr 2002/03 nach dem Vorbild der US-amerikanischen CDC auf Ebene der Europäischen Union als Koordinierungs- und Beratungsgremium eingerichtet wurde[36]. Seine Kernaufgabe ist es, aktuelle und neu auftretende Bedrohungen durch Infektionskrankheiten zu ermitteln, zu bewerten und entsprechende Informationen darüber weiterzugeben[37]. Zu diesem Zweck sammelt, erhebt und wertet das ECDC insb. einschlägige wissenschaftliche und technische Daten aus, stellt wissenschaftliches Fachwissen bereit und übernimmt die Koordination der Überwachungs- und Expertennetzwerke[38].

Anders als in Deutschland erfolgt dies in der Praxis bereits seit Jahren mit Hilfe mehrerer IT-Tools, mit denen innerhalb des Netzes zur epidemiologischen Überwachung Daten bzgl. Infektionskrankheiten und anderen Gesundheitsgefahren gesammelt und auswertet sowie Information zwischen Experten ausgetauscht werden. Im Mittelpunkt steht dabei die im Juni 2021 gelaunchte von der ECDC betriebene European Surveillance Plattform *EpiPulse*, die mehrere bis dato voneinander getrennte webbasierte Surveillance Systeme, wie etwa das European Surveillance Tool *TESSy* oder das Epidemic Intelligence Information System *EPIS* miteinander verknüpft[39]. Während *EPIS* die Funktion einer Kommunikationsplattform[40] innehatte, die den Informationsaustausch zwischen speziell benannten Gesundheitsexperten zur frühzeitigen Detektion von Gesundheitsbedrohungen erlaubt; diente *TESSy* in erster Linie als Datenbank und Schnittstelle zur Übermittlung und Nutzung von europaweiten Surveillance-Daten[41]. Die Daten entstammen dabei den zuständigen nationalen Behörden der Mitgliedstaaten, die diese entsprechend ihrer nationalen Vorgaben eigenverantwortlich erheben, sammeln und gemäß Art. 6 Abs. 3 des Beschlusses Nr. 1082/2013/EU sowie Art. 4 der VO (EG) Nr. 851/2004 mindestens einmal wöchentlich anhand von festgelegten Falldefinitionen[42] und Standardvariablen anonymisiert und – je nach Verfügbarkeit im eigenen Land – einzelfallbasiert oder auch aggregiert via *EpiPulse* übermitteln[43]. Besonders die COVID-19-Pandemie hat gezeigt, dass hier noch erhebliche Defizite, vor allem was die Vergleichbarkeit und

28) *Diercke et al.*, Bundesgesundheitsbl. 2021,389.
29) BT-Dr. 19/23944, S. 29; vgl. zudem *Diercke et al.*, Bundesgesundheitsbl. 2021,389 f.
30) COM (2020) 727 endg.; vgl. zur Kritik *Müller*, Europa und die Pandemie. Zuständigkeitsdefizite und Kooperationszwänge in: Staat und Gesellschaft in der Pandemie, 2021, S. 129.
31) ABl. L 293 v. 5. 11. 2013.
32) Auch wenn Art. 168 Abs. 5 AEUV von Fördermaßnahmen spricht, spricht die Tatsache, dass diese in einem ordentlichen Gesetzgebungsverfahren (Art. 289 Abs. 1 AEUV) zu erlassen sind, für die Annahme verbindlicher Rechtsakte, vgl. dazu *Berg/Augsberg*, in: *Schwarze/Becker/Hatje/Schoo*, EU-Kommentar, 4. Aufl. 2019, Art. 168 AEUV, Rdnr. 33.
33) *Berg/Augsberg*, in: *Schwarze/Becker/Hatje/Schoo*, EU-Kommentar, 4. Aufl. 2019, Art. 168 AEUV, Rdnr. 15; *Gassner*, in: *Kluckert*, Das neue Infektionsschutzgesetz, 2. Aufl. 2021, § 1, Rdnr. 85.
34) *Müller*, Europa und die Pandemie. Zuständigkeitsdefizite und Kooperationszwänge in: Staat und Gesellschaft in der Pandemie, 2021, S. 108; *Berg/Augsberg*, in: *Schwarze/Becker/Hatje/Schoo*, Art. 168 AEUV, Rdnr. 16; *Frenz*, Europarecht, 3. Aufl. 2021, Rdnr. 1130.
35) *Dąbrowska-Kłosińska*,: EJRR 2019, 652, 661.
36) Art. 1 der Verordnung (EG) Nr. 851/2004 des Europäischen Parlaments und des Rates v. 21. 4. 2004 zur Einrichtung eines europäischen Zentrums für die Kontrolle von Krankheiten, ABl. L 142 v. 30. 4. 2004.
37) Art. 3 der Verordnung (EG) Nr. 851/2004; s. weiterführend *Winkelmüller*, in: *Eckart/Winkelmüller*, IfSG, § 5, Rdnrn. 31 ff.; *Trute*, GSZ 2018, 125, 129.
38) Art. 3 Abs. 2 der Verordnung (EG) Nr. 851/2004.
39) https://www.ecdc.europa.eu/en/publications-data/epipulse-european-surveillance-portal-infectious-diseases (zuletzt abgerufen am 3. 8. 2022).
40) *EPIS* beinhaltete fünf verschiedene Krankheitscluster adressierende Plattformen, u. a. FWD (Food- and Waterborne Disease and Zoonoses), ELDsNet (European Legionnaires' Disease Surveillance Network), AMR-HAI (Antimicrobial Resistance and Healtcare-associated Infections), STI (Sexually Preventable Diseases) sowie VPD (Vaccine Preventable Diseases), https://www.ecdc.europa.eu/en/publications-data/epidemic-intelligence-information-system-epis (zuletzt abgerufen am 3. 8. 2022).
41) *Dąbrowska-Kłosińska*, EJRR 2019, 652, 661; Report on the implementation of Decision No 1082/2013/EU of the European Parliament and of the Council, p. 7.
42) Vgl. hierzu Anhang II des Durchführungsbeschlusses (EU) 2018/945, ABl. L 170/1 v. 6. 7. 2018.
43) *Dąbrowska-Kłosińska*, EJRR 2019, 652, 661; *Ammon/Faensen*, Bundesgesundheitsbl. 2009, 179.

Kompatibilität der übermittelten Datensätze und damit die Möglichkeiten der Wissensgenerierung aus diesen Daten betrifft, bestehen[44]. Da die Datenerhebung Sache der Mitgliedsstaaten ist, können Qualität und Detailgrad der übertragenen Daten je nach Datenerfassung(-spolitik) aber auch je nach Kapazität des jeweiligen Gesundheitssystems des Mitgliedstaates (bspw. was die Laborkapazitäten betrifft) varriieren und damit insgesamt die Fähigkeit der EU auf Basis dieser Daten grenzüberschreitende Gesundheitsgefahren rechtzeitig zu erkennen und darauf reagieren zu können, begrenzen. Zumal es in einigen Ländern schon an den entsprechenden gesetzlichen Grundlagen, um die europäischen Maßgaben zu unterstützen, fehlt[45]. So übermittelten bspw. in der Corona-Pandemie nicht alle Länder ihre Corona-Fälle aufgeschlüsselt nach Alter und Geschlecht; andere machten keine näheren Angaben zu ihren (unterschiedlichen) Teststrategie und -konzepten, was unmittelbaren Einfluss auf die Anzahl der bestätigten Fälle und Todeszahlen hatte[46].

Ergänzend zu diesem indikator-basierten Surveillance-Informationsmanagementsystem, das den direkten und horizontalen Daten- und Informationsaustausch zwischen den verantwortlichen nationalen und europäischen Akteuren ermöglicht, greift das ECDC zur Überwachung zunehmend auch auf inoffizielle Online-Daten basierende intelligente Surveillance-Instrumente (sog. event-based Surveillance) zurück, wie etwa das Global Public Health Intelligence Network (*GPHIN*), die von der Harvard Medical School entwickelte *HealthMap* oder das Medical Information System *MedISys*[47]. Allen Anwendungen ist dabei gemein, dass sie mit Hilfe von Algorithmen automatisch frei zugängliche Online-Quellen, wie bspw. Medienberichte oder Social-Media-Seiten, auf Hinweise zu Krankheitsausbrüchen scannen und analysieren mit dem Ziel potentielle Gefahren für die europäische Gesundheit noch frühzeitiger erkennen zu können. Nur das Media Monitoring System *MedISys* ist dabei ein originär Europäisches. Es wurde 2004 von der Gemeinsamen Forschungsstelle der EU-Kommission etabliert und hat seither in zahlreichen Fällen Warnmeldungen für Gesundheitsnotfälle, wie etwa beim Ausbruch der Schweinegrippe 2009, abgegeben[48].

Zugang zu den von den verschiedenen Surveillance-Systemen gesammelten Daten erhalten dabei allerdings bislang vorwiegend nur assoziierte Experten. Während zwar die aus den Daten gewonnenen Erkenntnisse, wie es auch der Aufgabe des ECDC entspricht, in Berichten, HeadMaps etc. für jedermann zugänglich sind, bleiben vor allem die von den europäischen Surveillance-Tools wie *EpiPulse* generierten Datasets vorwiegend den von den Mitgliedstaaten benannte Experten sowie Vertretern der europäischen Behörden und internationalen Organisationen vorbehalten[49]. Externe Forschern haben lediglich die Möglichkeit aggregierte Daten oder Teilmengen davon zu erhalten, was deren Nutzbarmachung etwa für weitere Forschungszwecke bspw. in der Bioinformatik beschränkt.

#### *2. Digitalisierung der Surveillance – Der Vorschlag zur Gesundheitsunion*

Mit dem Vorschlag zur Gesundheitsunion[50], der sich in eine Reihe weiterer Digitalstrategien[51] der EU einordnet, soll das beschriebene europäische Surveillance-System vor allem durch einen verstärkten Einsatz digitaler Lösungen (wie KI, Hochleistungsrechner, Computermodellierungen oder Simulationssysteme) sowie durch Erweiterung der dafür heranzuziehenden Datenquellen modernisiert und weitergehend digitalisiert werden. Kam bislang keinerlei BigData zur Analyse bei den vom ECDC eingesetzten Systemen zur Anwendung[52], will sich die EU diese in Zukunft sowohl im Rahmen der epidemiologischen Überwachung als auch im Rahmen des Frühwarn- und Reaktionssystems (Early Warning and Response System, EWRS) zu Nutze machen, wobei die konkrete Ausgestaltung – wie auch zuvor – den verantwortlichen Behörden überlassen bleibt.

##### a) Einsatz intelligenter Systeme

Im Mittelpunkt des Vorschlags steht dabei die Weiterentwicklung der digitalen Surveillance-Plattform, deren Existenz und die an sie zu stellenden (technischen) Anforderungen damit erstmals kodifiziert und beschrieben werden würden[53]. Denn bis dato liegt die technische Ausgestaltung der Infrastruktur im Ermessen der verantwortlichen europäischen und mitgliedstaatlichen Behörden, auch weil der bisherige Rechtsrahmen[54] relevante Begriffe (wie „Netz“, „System“, „Übermittlung von Informationen“) nicht näher konkretisiert[55]. Gemeint sein dürfte mit der digitalen Surveillance-Plattform vor allem die 2021 gelaunchte Plattform *EpiPulse* bzw. deren potentielle Nachfolger, über die die Mitgliedstaaten sowohl ihre Surveillance Daten als auch Expertenwissen miteinander austauschen und die damit zur Säule des Europäischen Surveillance Systems avancieren dürfte. Nach dem Vorschlag zur Gesundheitsunion soll diese nun in Zukunft nicht nur wie bisher die computergestützte Verwaltung und den computergestützten Austausch von Informationen, Daten und Dokumenten ermöglichen, sie soll darüber hinausgehend auch die automatisierte Erhebung von Surveillance- und Labordaten sowie die Nutzung Künstlicher Intelligenz zur Validierung, Analyse und automatisierten Meldung erlauben[56]; wobei die EU-Kommission Einzelheiten zu den technischen Anforderungen an die Plattform sowie zum Datenschutz noch in Durchführungsrechtsakten regeln will. Angestrebt wird also der Aufbau eines intelligenten zentralen Überwachungssystems, welches Surveillance Daten nicht nur automatisch erhebt, sondern auch mit Hilfe von BigData automatisch auf Vorkommnisse analysiert und dadurch eine präzisere Überwachung und Risikobewertung in Echtzeit ermöglicht.

Neben der Modernisierung des Surveillance-Systems soll zudem das Frühwarn- und Reaktionssystem (EWRS), mit dem Warnmeldungen über schwerwiegende grenzüberschreitende Gesundheitsgefahren rasch übermittelt werden können, durch intelligente Hilfsmittel ergänzt werden. So soll etwa mit Hilfe moderner Technologien (wie digitale mobile Anwendungen, Modelle künstlicher Intelligenz oder weltraumgestützte Anwendungen) und aufbauend auf den von den Mitgliedstaaten entwickelten Technologien

44) COM (2020) 724 final, S. 17; *Renda/Castro*, EJRR 2020, 278.
45) Vgl. hierzu *Speakman/Burris/Coker*, Pandemic Legislation in the European Union: Fit for Purpose? The Need for a Systematic Comparison of National Laws (2017) 121 Health Policy 1021.
46) Vgl. *Renda/Castro*, EJRR 2020, 273, 278.
47) Zu den verschiedenen Systemen vgl. *Roberts*, EJRR 2019, 722, 729 ff.
48) *Roberts*, EJRR 2019, 722, 771.
49) Für die Vorgänger-Systeme TESSy und EPIS vgl. *Dąbrowska-Kłosińska*, EJRR 2019, 652, 661 f.
50) Vgl. Vorschlag für eine Verordnung des Europäischen Parlaments und des Rates zu schwerwiegenden grenzüberschreitenden Gesundheitsgefahren und zur Aufhebung des Beschlusses Nr. 1082/2013/EU, COM (2020) 727 final sowie COM (2020) 724 final.
51) Genannt seien hier nur der Vorschlag für einen Artificial Inteligance Act oder den Data Gouvernance Act (COM (2020) 767 final) genannt.
52) Vgl. Third independent external evaluation of the ECDC, 2019, p. 93, https://www.ecdc.europa.eu/sites/default/files/documents/third-independent-external-evaluation-of-ECDC-report.pdf (zuletzt abgerufen am 3. 8. 2022).
53) Vgl. Art. 14 COM (2020) 727 final.
54) Beschluss Nr. 1082/2013/EU, ABl. L 293/9 v. 5. 11. 2013.
55) *Dąbrowska-Kłosińska*, EJRR 2019, 652, 659.
56) Art. 14 Abs. 2 COM (2020) 727 final.

ein automatisiertes System zur (grenzüberschreitenden) Kontaktverfolgung eingerichtet werden[57] – habe sich doch in der Corona-Pandemie die Wichtigkeit solcher Tools erwiesen. Die Verantwortung soll auch hierfür das ECDC tragen, wobei es dafür wohl einer deutlichen Aufstockung seiner Kapazitäten bedürfte; sind letztere doch mit bislang 300 Mitarbeitern und 60 Mio. EUR Jahresbudget eher begrenzt[58].

b) Erweiterung der Datenlage und Verbesserung der Vergleichbarkeit

Jenseits der Nutzung intelligenter Methoden zur Datenerhebung und -analyse strebt der Vorschlag zur Gesundheitsunion auch die Einbeziehung neuer Datenquellen, die über die herkömmliche indikatorbasierte Surveillance anhand von Fall- und Labordaten etc. hinausgehen, an, wodurch insgesamt eine Erweiterung der Erkenntnisquellen erreicht werden soll. So will die EU zukünftig Abwasserdaten, Erdbeobachtungs- und Geolokalisationsdaten aus dem Weltraum oder explizit auch Informationen aus Medienbeobachtungen zur Überwachung nutzen[59]. Zudem sollen zukünftig auch Daten aus elektronischen Gesundheitsakten in die Surveillance-Systeme einfließen[60]. Die genaue Umsetzung und Ausgestaltung bleibt dabei – wie so vieles bei dem Vorschlag – noch ungewiss, zumal die unterschiedlichen Entwicklungsstadien was die Digitalisierung der Gesundheitssysteme in den einzelnen Ländern betrifft sowie die vielfach fehlende Infrastruktur die verantwortlichen Behörden dabei vor große Herausforderungen stellen dürfte. Denn während die elektronische Patientenakte bspw. in Schweden bereits flächendeckend eingeführt ist, steckt sie hierzulande noch in den Kinderschuhen[61]. Datenqualität und -umfang dürften daher also je nach Mitgliedstaat divergieren; abgesehen davon, dass die Daten bspw. hierzulande gar nicht von den zur Übermittlung verpflichteten nationalen Behörden, sondern von den Krankenkassen verwaltet werden, vgl. § 341 SGB V.

In jedem Fall wird sich der Gebrauch der aus den Gesundheitsakten stammenden Daten aufgrund des begrenzten Mandats der EU in diesem Bereich nur auf von zur Bekämpfung grenzüberschreitender Gesundheitsgefahren einschlägiger Daten beschränken müssen. Da es sich bei Daten aus Gesundheitsakten regelmäßig um sensible Gesundheitsdaten i. S. v. Art. 4 Nr. 15 DSGVO handeln wird, können diese zu Public Health-Zwecken nur auf Grundlage einer Rechtsgrundlage und unter Wahrung des Verhältnismäßigkeitsgrundsatzes an das ECDC übermittelt werden (Art. 9 Abs. 2 lit. i) DSGVO)[62]. Zwar liegt eine solche Rechtsgrundlage mit dem Beschluss Nr. 1082/2013/ EU bzw. der geplanten NachfolgeVO vor, ihr Anwendungsbereich beschränkt sich jedoch ausschließlich auf die Bewältigung schwerwiegender grenzüberschreitender Gesundheitsgefahren (vgl. jeweils Art. 1), womit ein Gebrauchmachen darüberhinausgehender Gesundheitsdaten – etwa zur allg. Vermessung der Gesundheit der europäischen Bevölkerung – jedenfalls auf dieser Grundlage ausgeschlossen bleibt.

Zusätzlich soll mit der Einrichtung von EU-Referenzlaboratorien[63] schließlich noch dem in der Pandemie zutage getretenen Problem fehlender vergleichbarer Surveillance-Daten aus den unterschiedlichen Mitgliedsstaaten entgegengewirkt werden[64]. Zwar existierte bereits eine verbindliche Liste mit Falldefinitionen, epidemiologischen und klinischen Kriterien der überwachten Krankheiten[65], die die Vergleichbarkeit und Komptabilität der Daten sicherstellen soll, gleichwohl hängt dies letztendlich entscheidend von der jeweilig eingesetzten Labordiagnostik und den Testmodalitäten vor Ort ab. Entsprechend sieht der Vorschlag die Möglichkeit der Unterstützung nationaler Referenzlaboratorien durch von der ECDC koordinierte EU-Referenzlaboratorien vor, um eine Angleichung der nationalen Diagnostik und Testmethoden zur einheitlichen Überwachung und Meldung von Krankheiten auf freiwilliger Basis zu erreichen[66]. Eine verpflichtende Vereinheitlichung scheidet aufgrund der beschriebenen, auf vornehmlich Unterstützungsmaßnahmen begrenzte Unionskompetenz aus.

### IV. Schluss

Sowohl auf nationaler, viel mehr aber noch auf europäischer Ebene kommen inzwischen eine Vielzahl unterschiedlicher – häufig separat agierender – digitaler Surveillance-Systeme zum Einsatz, die eine kontinuierliche Wissensbasis bzgl. der Entstehung, Ausbreitung und Folgen übertragbarer Krankheiten für die Verantwortungsträger bereitstellen sollen. Umfang, Zweck und Grad der Digitalisierung der jeweils eingesetzten Systeme variieren dabei erheblich und reichen von einfachen Datenverarbeitungssystemen bis hin zu automatisierten, mit Hilfe von BigData-Analyseverfahren agierenden Tools.

Vor allem die EU-Kommission treibt nach den Erfahrungen aus der Covid-19-Pandemie mit ihrem Vorschlag zur Gesundheitsunion dabei die KI-gestützte Surveillance der infektionsepidemiologischen Überwachung auf europäischer Ebene voran, während Deutschland noch am Aufbau einer digitalen Infrastruktur, die das nötige Wissen zur Überwachung und Steuerung generiert, arbeitet. Vor allem föderale Hemmnisse, die Vielzahl der unterschiedlichen im Gesundheitswesen aktiven Akteure, die es einzubinden gilt, aber auch das kulturell eher schwierige Verhältnis der Deutschen zur Datensammlung, wirken hier erschwerend[67]. Dabei wäre ein schnelles Vorankommen in dieser Sache nicht nur mit Blick auf die Legitimation künftiger Public Health Maßnahmen anhand einer exakteren Datenlage notwendig. Der Aufbau einer zeitgemäßen, mit digitalen Surveillance Instrumenten ausgestatteten Wissensinfrastruktur ist auch angesichts der Erwartung, dass sich derartige Bedrohungslagen in einer globalisierten Welt wie der heutigen künftig häufen könnten, unerlässlich[68]. Das Melde- und Informationssystem DEMIS kann dabei langfristig ein Baustein sein, wichtiger aber wären noch verpflichtend einzuhaltende Datenformate und -standards sowie das Vorhalten technischer Schnittstellen, die eine

57) Art. 18 Abs. 2 COM (2020) 727 final; bislang war die Kontaktnachverfolgung lediglich eines der Elemente, die die Mitgliedstaaten über das EWRS koordinieren sollten, vgl. COM (2020) 724 final, S. 20.

58) *Anderson/McKee/Mossialos*, Covid-19 exposes weaknesses in European response to outbreaks, 2020, 368 BMJ m1075; *Renda/Castro,* EJRR 2020, 273, 277; vgl. auch *Greer,* JHPPL 2012, 1001, 1002.

59) COM (2020) 724 final, S. 17 f.; Medienbeobachten erfolgen bereits heute schon fortlaufend etwa mithilfe des seit 2004 existieren Informationssystems *MedISys*, s. bereits unter III. 2.

60) Vgl. Art. 14 Abs. 2a COM (2020) 727 final; COM (2020) 724 final, S. 17.

61) *Carlsson*, DÄBl. 2016; 113 (39), A-1703.

62) Vgl. auch ErwG 22 zu COM (2020) 727 final.

63) Vgl. Art. 15 COM (2020) 727 final.

64) S. hierzu unter III. 1.

65) Vgl. Anhang II des Durchführungsbeschlusses (EU) Nr. 2018/2945 der Europäischen Kommission v. 22. 6. 2018 über die durch epidemiologische Überwachung zu erfassenden übertragbaren Krankheiten und damit zusammenhängenden besonderen Gesundheitsrisiken sowie über die entsprechenden Falldefinition, ABl. L 170/1 v. 6. 7. 2018.

66) Vgl. Art. 15 Abs. 1 COM (2020) 727 final.

67) Hier bedarf es jedoch noch weitergehender Forschung.

68) Vgl. dazu etwa Rede von Kommissionspräsidentin *Ursula von der Leyen* beim „One Planet Summit 2021“ in Paris, wo sie von einer möglicherweise bevorstehenden „era of pandemics“ spricht, https://ec.europa.eu/commission/presscorner/detail/en/speech_21_61 (zuletzt abgerufen am 3. 8. 2022).

Zusammenführung der bereits in unterschiedlichen Systemen generierten Daten zu Analysezwecken zulassen[69].

**Open Access.** Dieser Artikel wird unter der Creative Commons Namensnennung 4.0 International Lizenz veröffentlicht, welche die Nutzung, Vervielfältigung, Bearbeitung, Verbreitung und Wiedergabe in jeglichem Medium und Format erlaubt, sofern Sie den/die ursprünglichen Autor(en) und die Quelle ordnungsgemäß nennen, einen Link zur Creative Commons Lizenz beifügen und angeben, ob Änderungen vorgenommen wurden.
Die in diesem Artikel enthaltenen Bilder und sonstiges Drittmaterial unterliegen ebenfalls der genannten Creative Commons Lizenz, sofern sich aus der Abbildungslegende nichts anderes ergibt. Sofern das betreffende Material nicht unter der genannten Creative Commons Lizenz steht und die betreffende Handlung nicht nach gesetzlichen Vorschriften erlaubt ist, ist für die oben aufgeführten Weiterverwendungen des Materials die Einwilligung des jeweiligen Rechteinhabers einzuholen.
Weitere Details zur Lizenz entnehmen Sie bitte der Lizenzinformation auf http://creativecommons.org/licenses/by/4.0/deed.de.

69) Vgl. dazu jüngst das Positionspapier des Wissenschaftsrats, Digitalisierung und Datennutzung für Gesundheitsforschung und Versorgung, Juli 2022, S. 44 ff.

## Perspektiven der molekulargenetischen Therapien

### Zur Notwendigkeit der Weiterentwicklung des AMNOG Bewertungssystems[1] im Bereich der personalisierten Medizin

**Silvia Woskowski* und Claus Burgardt**

#### Einleitung

Im Zeitalter der personalisierten Medizin führen hochspezialisierte Technologien gemeinsam mit der Digitalisierung und dem Wissen über die Genetik zu einer Beflügelung des Präzisionsgrades bei der Wirksamkeit und Verträglichkeit neuer Therapien. Dabei hat für die Behandlung vieler Krankheiten eine neue Ära begonnen, in der durch Next Generation Sequencing (NGS) genetische Informationen gewonnen werden und die Grundlage individueller Präventions- und Therapieentscheidungen bilden. Hierbei kann z. B. mit der NGS basierten Einzelzellsequenzierung in die Zellen eines Gewebes hineingeschaut und ein digitales Bild des molekularen Zustandes erstellt werden. Auf diese Weise lassen sich frühe molekulare Veränderungen erforschen[2]. Und auch in der Behandlung zeigen sich neue Perspektiven durch molekulargenetische Therapien. Zum Beispiel mit sog. Tumoragnostischen Behandlungsansätzen und dem Einsatz der mRNA-Technologie in der Onkologie. Sie sind die Grundlage neuer personalisierter Therapien zur Behandlung onkologischer Erkrankungen. Um einen generellen Zugang zu einer sektorenübergreifenden, individualisierten Versorgung zu ermöglichen und die Forschung in diesem Bereich weiter voranzutreiben, benötigen wir mehr Gesundheitsdaten, die vertrauensvoll und sicher erhoben, verarbeitet sowie zugunsten und im Sinne der Versicherten genutzt werden. Dies gilt insbesondere dann, wenn es um den Nachweis genetischer Veränderungen, z. B. bei der molekulargenetischen Diagnostik oder die Sequenzierung des Genoms bei erblich bedingten Erkrankungen geht. Erste Schritte in diese Richtung regelt das in § 64e SGB V normierte „Modellvorhaben zur umfassenden Diagnostik und Therapiefindung mittels Genomsequenzierung bei seltenen und onkologischen Erkrankungen“[3]. Es wird voraussichtlich 2024 beginnen und mindestens fünf Jahre mit dem Ziel der Übernahme in die Regelversorgung laufen[4]. Geschaffen werden sollen Qualitätsstandards für den klinischen Einsatz von Genomdiagnostik, qualitätsgesicherte Sequenzierungen und die interdisziplinäre, wissenschaftliche Auswertung der Daten, um Patienten personalisiert zu behandeln.

#### I. Ausgangslage

In der Onkologie ist die personalisierte Medizin besonders weit. Durch eine Analyse der Tumor-DNA werden hier individuelle genetische Veränderungen diagnostiziert, über die sich die Patienten identifizieren lassen, die von einer personalisierten Krebstherapie profitieren können (sog. Tumoragnostischer Behandlungsansatz). Im Zentrum stehen dabei heute nicht mehr die Organe, in denen der Tumor auftritt, sondern die individuellen Genomveränderungen, die das Krebswachstum verursachen und antreiben. Durch die Möglichkeiten, diese individuellen Merkmale des Tumors zu ermitteln, nimmt sachlogisch die Heterogenität der Patientengruppen zu und die Größe der Patientenkollektive ab. Bei der Entwicklung neuer Arzneimittel (AM) ist dabei die Durchführung randomisierter kontrollierter klinischer Studien (sog. RCT)[5] z. T. bereits heute unmöglich. Dies führt zu veränderten Studiendesigns

Rechtsanwältin Dr. iur. Silvia Woskowski, LL.M.,
Anklamer Straße 32, 10115 Berlin, Deutschland

Rechtsanwalt Claus Burgardt, STRÄTER Rechtsanwälte PartmbB,
Kronprinzenstraße 20, 53173 Bonn, Deutschland

*) Die Autorin befasst sich schwerpunktmäßig mit rechtichen und gesellschaftspolitischen Fragestellungen der Präzisionsmedizin und Digitalisierung im nationalen und europäischen Kontext.

1) Der verwendete Oberbegriff umfasst im Folgenden Nutzenbewertung und Preisregulierung. Nach dem Willen des Gesetzgebers besteht das Regulierungskonzept aus zwei Verfahrensabschnitten. Der Nutzenbewertung des Arzneimittels mit dem neuen Wirkstoff durch den Gemeinsamen Bundesausschuss (G-BA) und der Preisregulierung durch die Verhandlung bzw. Festsetzung des Erstattungsbetrages (BT-Dr. 17/2413, S. 19 f.).

2) Beispielhaft für die voranschreitende Personalisierung ist auch die S3-Leitlinie zur Behandlung des kolorektalen Karzinoms (AWMF-Registernummer 021/0070 L). Sie sieht Maßnahmen zur initialen molekularbiologischen Diagnostik im Vorfeld der Therapieeinleitung vor.

3) Implementiert mit dem Gesundheitsversorgungsweiterentwicklungsgesetz (GVWG) v. 11. 7. 2021, BGBl. I S. 2754.

4) Grds. ist vorgesehen, bereits ab Januar 2023 zu starten. Da die Konzipierung der erforderlichen Dateninfrastruktur bis dahin unwahrscheinlich ist, hat man den Beginn entsprechend adaptiert.

5) Randomized Controlled Trials.

Funding

Open access funding enabled and organized by Projekt DEAL.

